# Sensitivity and characteristics associated with positive QuantiFERON-TB Gold-Plus assay in children with confirmed tuberculosis

**DOI:** 10.1371/journal.pone.0213304

**Published:** 2019-03-04

**Authors:** Duc T. Nguyen, Ha Phan, Trang Trinh, Hang Nguyen, Ha Doan, Nam Pham, Hung Nguyen, Hanh Nguyen, Hung V. Nguyen, Hoi V. Le, Nhung Nguyen, Edward A. Graviss

**Affiliations:** 1 Houston Methodist Research Institute, Houston, TX, United States of America; 2 Center for Promotion of Advancement of Society (CPAS), Lot A4 15 Dong Quan, Cau Giay, Ha Noi, Vietnam; 3 Vietnam National Tuberculosis Program/University of California San Francisco Research Collaboration, Ha Noi, Vietnam; 4 National Lung Hospital, Hoang Hoa Tham, Ba Dinh District, Ha Noi, Vietnam; National Institute for Infectious Diseases (L. Spallanzani), ITALY

## Abstract

**Background:**

Although QuantiFERON-TB Gold Plus (QFT-Plus), a new interferon-gamma release assay, has shown good performance in adults, little data is available in children.

**Methods:**

De-identified data from TB-suspected patients age <18 years with QFT-Plus results, who were admitted or screened at the National Lung Hospital (NLH) in Ha Noi, Vietnam in 2017, were assessed. Logistic regression analyses were performed to determine the characteristics associated with having a positive QFT-Plus result. Sensitivity, both overall and in subgroups of pulmonary TB only (PTB), extra-pulmonary TB (EPTB) only, and both PTB and EPTB were calculated.

**Results:**

Of 222 children with available QFT-Plus results, 33 were classified as confirmed TB, of whom 18 had QFT-Plus (+) and 15 had QFT-Plus (-). Multiple logistic regression modeling suggested that age, history of TB, and confirmed TB were significantly associated with having a positive QFT-Plus result with an area under the ROC curve of 0.77. QFT-Plus sensitivity in PTB only, EPTB, and both PTB and EPTB patients was 84.2%, 14.3% and 14.3%, respectively. The overall sensitivity of the QFT-Plus assay (regardless PTB or EPTB) in children was 54.5%.

**Conclusion:**

Although QFT-Plus had a good sensitivity in children having exclusive PTB, it had poor sensitivity in EPTB.

## Background

Despite numerous efforts by tuberculosis (TB) control programs worldwide to control the disease in adults, pediatric TB has not received adequate attention [1]. As such, pediatric TB remains a major public health issue with at least one million children newly identified with TB each year, representing 10–11% of all TB cases [2, 3]. The World Health Organization (WHO) recently reported an estimate of 250,000 deaths among pediatric TB cases, of whom an estimated 50,000 were children living with HIV infection [4]. Obtaining a definitive TB diagnosis in children is challenging for clinicians due to the lack of specific signs and symptoms consistent with TB, difficulty in obtaining sputum samples in children and the paucibacillary nature of *Mycobacterium tuberculosis* (*Mtb*) in the clinical specimens [5]. Additionally, treating childhood TB is hindered by the unavailability of pediatric formulations, drug toxicity, and treatment compliance [6–8]. Therefore, early detection and treatment of tuberculosis infection (TBI) to prevent the development of TB disease in children are important strategies to achieve TB elimination goals [9–11]. In adults, 5% to10% of the infected individuals will develop TB disease with 50% of the cases occurring in the first two years after infection [12]. The risk of TB progression is much higher in children than in adults with 30–40% of infants having TB infection progressing to develop TB disease and 10–20% of TB-infected children developing pulmonary disease in the second year of life [13]. Therefore, accurate diagnostic tests which can detect TBI early would be effective tools for TB programs in controlling TB disease in children.

A blood test first introduced in 2015 which can detect *Mtb* infection, QuantiFERON-TB Gold Plus (QFT-Plus, QIAGEN, Germantown, MD, USA) is an interferon-gamma (IFN-γ) release assay (IGRA). Different from its predecessor, QuantiFERON-TB Gold In-Tube (QFT-Gold), QFT-Plus has no TB7.7 peptides in its antigenic components. Instead, QFT-Plus has a second antigen tube (TB2) containing shorter peptides for ESAT-6 and CFP-10, which aims at eliciting a response from CD8+ T-cells in addition to the peptides directed at CD4+ cells in the first antigen tube (TB1). Additionally, QFT-Plus allows direct in-tube phlebotomy or indirect phlebotomy into a lithium heparin (LiHp) tube, where the blood is subsequently transferred into the four QFT-P tubes in the laboratory. The later phlebotomy method may reduce indeterminate results. The QFT-Plus assay is considered to have higher sensitivity than that of QFT-Gold which has an overall sensitivity of 94.1% and a specificity of 97.3% in adults [14]. Given that no gold standard for latent TBI (LTBI) diagnosis is available, there have been studies of the diagnostic performance of QFT-Plus in active TB populations to evaluate the assay’s sensitivity [10, 15–17]. However, current literature related to the QFT-Plus shows that studies were conducted mainly in adults. Information regarding the sensitivity and specificity of the QFT-Plus in children is still limited. For that reason, our study attempted to (1) identify characteristics that are associated with a positive QFT-Plus result in children in a high TB prevalence setting, and (2) calculate the sensitivity of QFT-Plus in children having TB disease.

## Methods

The study was part of the Zero Tuberculosis Initiative (https://www.zerotbinitiative.org/) conducted at the National Lung Hospital (NLH) in Ha Noi to inform health care providers and policy makers with the goal of finding more effective diagnostic tools for TB infection in children within Vietnam. The study used de-identified data from a retrospective medical chart review for all patients age <18 years who were consecutively admitted to the Department of Pediatrics at the NLH during the year 2017 and had the QFT-Plus results available in their medical charts. Located in Ha Noi, Vietnam, the NLH is a 600-bed TB referral hospital for the northern region of Vietnam. During the study time frame in 2017, there were 259 pediatric TB cases being diagnosed at the NLH, of whom 85 were confirmed by *Mtb* culture. Approximately 80% of the patients seen at the NLH are referred from other health facilities. Since April 2017, the QFT-Plus assay has been used as an optional TB screening test for children suspected of having TB who come to be evaluated by physicians at the NLH. As the QFT-Plus is not part of the Vietnamese National TB Program, the cost of the assay is paid for by the patients or their parents. The NLH physicians introduced the availability of QFT-Plus assay and its potential benefits and inconveniences to the patient and family during their first visit. The physicians also emphasized to parents the limited information on how the assay performs in pediatric populations and that choosing or refusing the use of QFT-Plus would not affect the standard of care for their child. Chart review was conducted by NLH medical staff. De-identified data were entered into an EpiData (Aarhus University, Denmark) database (via double-entry) which was specifically created for this study. As this was a retrospective study using de-identified data, ethical approval was not required by the NLH Institutional Review Board.

Inclusion criteria was defined as: (1) age <18 years; (2) consecutively seen by physicians at the NLH from April through December 2017; and (3) QFT-Plus results available in the medical chart. Patients who were ≥18 years of age and did not have QFT-Plus results were not included in this study. TB diagnosis categories were defined on the basis of the standardized guidelines for pediatric TB case definition described by Graham et al. as follows [18, 19]:

*Confirmed TB patients*: Defined as children having symptoms compatible with TB (such as persistent cough >2 weeks, weight loss or failure to thrive, persistent unexplained fever >38°C for more than 1 week and persistent, unexplained lethargy or reduced playfulness) [19], and a bacteriologic confirmation by either culture or GeneXpert MTB/RIF assay (Xpert) from at least one respiratory specimen. In this study, patients who had pathological results consistent to TB lesions from specimens taken from pulmonary or extrapulmonary biopsy, were also classified as confirmed TB.

*Unconfirmed TB patients*: Defined as children who did not have bacteriologic confirmation and had at least two of the following: signs or symptoms suggestive of TB, positive TB-CXR, close TB contact, favorable response to TB treatment, or immunologic evidence of *Mtb* infection. As QFT-Plus was being evaluated in this analysis, the assay was not used as a criterion for TB categories. In addition, TST is no longer used in Vietnam. Therefore, immunologic evidence” was not part of the diagnosis classification in this study.

*Unlikely TB patients*: Defined as children who had symptoms compatible with tuberculosis but did not have bacteriologic confirmation and did not meet the criteria for unconfirmed TB [18, 19].

Body mass index (BMI) was calculated from weight and height, and standardized for age and gender (BMI z-score) based on the WHO reference charts [20]. Chest radiography (CXR) findings were collected from the original CXR report from a well-trained, experienced radiologist at the NLH, who was blinded from the patient’s clinical diagnosis. Abnormalities consistent with TB disease on chest radiography (TB-CXR) were based on the radiographic criteria suggested by Marais et al. in their proposed radiological classification of childhood intra-thoracic TB and were recorded in the dataset as a binary variable (normal versus abnormal) [21].

In this study, extra-pulmonary TB (EPTB) was confirmed by a histology result suggestive for TB from a sample collected at an extra-thoracic lesion. Patients who had both sputum culture positive for *Mtb* and a positive histology result were classified as having both Pulmonary TB (PTB) PTB and EPTB.

Acid-fast bacilli (AFB) sputum smears were performed using the Ziehl-Neelsen (ZN) method. Sputum cultures were performed with liquid media using the Becton Dickinson BacTec MGIT 960 system. Xpert was performed on a GeneXpert Gx4 device. The QFT-Plus assay was performed on the BioTek ELx800 and ELx50 platforms.

### Statistical analysis

Demographic and clinical data were reported as median and interquartile range (IQR) for continuous variables and as frequencies and proportions for categorical variables. Differences in demographic and clinical characteristics between the TB categories were compared using the Kruskal-Wallis test for continuous variables and Chi-square or Fisher’s exact tests for categorical variable, as appropriate. Univariate and multiple logistic regression analyses were performed to determine the characteristics associated with having a positive QFT-Plus result. Variables having a p-value of <0.2 in the univariate analysis were investigated further by multivariate Cox proportional hazards modeling. Variable selection for the multiple logistic regression models was conducted using the Bayesian model averaging (BMA) method [22, 23]. The Likelihood Ratio test was used to reduce further the model sets. The final model was chosen based on the smallest Bayesian information criterion (BIC). Model discrimination was determined by the area under the Receiver Operating Characteristic (ROC) curve (AUC).

The overall sensitivity (95% confidence interval, CI) was calculated by the proportion of QFT-Plus (+) in children with confirmed TB. The QFT-Plus sensitivity was also calculated in the subgroups of TB site such as PTB only, EPTB only, and PTB + EPTB only. The similar stratification for the sensitivity was also performed for the subgroups of children ≤5 years old and 6–17 years old. All the analyses were performed with Stata version 14.2 (StataCorp LLC, College Station, TX, USA). A p value of <0.05 was considered statistically significant.

## Results

### Characteristics of the study sample

**Fig 1:**
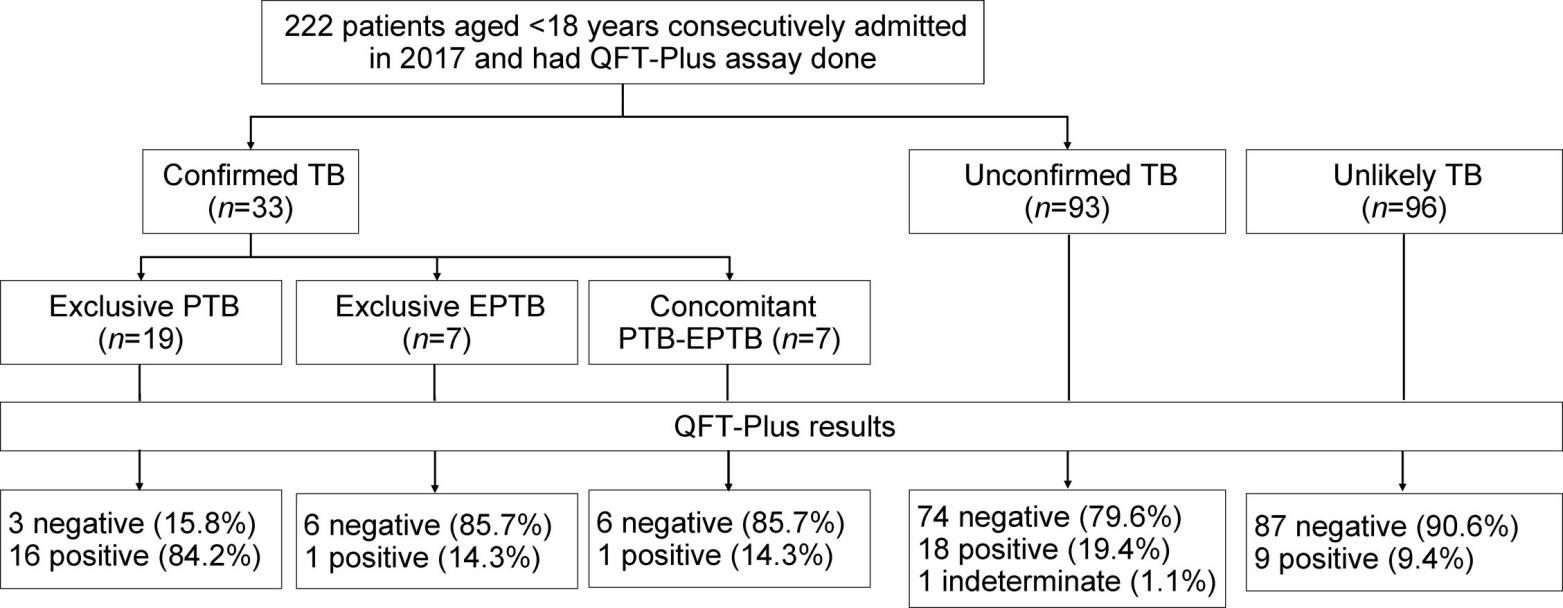
Flowchart of the study population. Footnote: TB, tuberculosis; QFT-Plus, QuantiFERON-TB Gold-Plus.

In 738 children admitted to the NLH Department of Pediatrics from April through December 2017, 222 had QFT-Plus results available. Of 33 patients classified as confirmed TB, 18 had QFT-Plus (+) and 15 had QFT-Plus (-). In the remaining 189 patients (unconfirmed or unlikely TB), there were 27 QFT-Plus (+), 161 QFT-Plus (-) and one indeterminate QFT-Plus results (Fig 1). Of note, 7 out of 96 (7.3%) unlikely TB patients had a QFT-Plus (+) result and would have met the criteria of the unconfirmed TB category if the QFT-Plus result were used as immunologic evidence for *Mtb* infection. In 96 patients classified as unlikely TB, 9 (9.38%) patients had QFT-Plus (+) with 5 (10.9%) aged ≤5 years and 4 (8.2%) aged 6–15 years.

The included children had an age range from 2 months to 16.8 years (median 5.2 years; IQR 2.2, 10.4), with nearly half of the children being ≤5 years of age (105/222, 47.3%). More than 25% of the included children had malnutrition with a BMI z-score <-2. The majority of the children (175/222, 78.8%) had at least one clinical sign or symptom consistent with TB and cough was the most common symptom (70.3%). Half of the children had positive TB radiography (TB-CXR). Only 27.5% of the children were tested for HIV, of whom none were known to have HIV infection. In total, 33/222 (14.9%) children were identified as confirmed TB by at least one of the 3 *Mtb* confirmation tests, culture, Xpert or tissue biopsy (24 patients had a single microbiologic result and 9 patients were confirmed with two microbiologic results). Of 33 patients with confirmed TB, 18 (54.6%) confirmed by a positive *Mtb* culture (including 9 having positive culture alone, 7 having both positive culture and Xpert, and 2 having both positive culture and histology), 2 (6.1%) confirmed by Xpert only, and 13 (39.4%) confirmed by histology only. Compared with unconfirmed or unlikely TB patients, confirmed TB patients were likely to be older (median age 9.8 years; IQR 2.3, 13.4 versus 5.2 years; IQR 2.4, 10.6, p = 0.009), have a significantly higher proportion of BMI z-scores ≤-2 (39.4% versus 23.0%, p = 0.046), and a longer length of stay (median 14.0 days; IQR 7.0, 17.0 versus 8.0 days; IQR 6.0, 12.0, p<0.001). The proportion of positive QFT-Plus results in unlikely, unconfirmed and confirmed TB patients was 9.4%, 19.4% and 54.5%, respectively (Table 1).

**Table 1:** Demographic and clinical characteristics of the study population.

Characteristics	Total	Unlikely TB	Unconfirmed TB	Confirmed TB	Overall
	(N = 222)	(*n* = 96)	(*n* = 93)	(*n* = 33)	p-value
**Demographics and anthropometric parameters**					
Age (years), median (IQR)	5.8 (2.4, 10.7)	5.5 (2.7, 10.7)	4.9 (1.8, 10.3)	9.8 (2.3, 13.4)	0.20
Age (years)					0.36
0–5	105 (47.3)	46 (47.9)	47 (50.5)	12 (36.4)	
6–10	48 (21.6)	22 (22.9)	20 (21.5)	6 (18.2)	
11–15	62 (27.9)	27 (28.1)	22 (23.7)	13 (39.4)	
16–17	7 (3.2)	1 (1.0)	4 (4.3)	2 (6.1)	
Male gender	134 (60.4)	59 (61.5)	58 (62.4)	17 (51.5)	0.53
BCG vaccination scar	218 (99.1)	94 (98.9)	91 (98.9)	33 (100.0)	0.84
Height (cm)	113.0 (85.0, 140.0)	112.0 (90.0, 138.0)	110.0 (80.0, 138.0)	130.0 (90.0, 150.0)	0.16
Weight (kg)	18.0 (11.0, 31.0)	18.0 (12.0, 30.0)	17.5 (10.0, 29.5)	23.0 (11.0, 36.0)	0.52
BMI (raw)	15.6 (13.7, 18.1)	16.0 (13.8, 18.2)	16.0 (14.2, 18.2)	15.0 (13.0, 16.6)	0.15
BMI z-score for sex and age, median (IQR)	-0.6 (-2.0, 0.8)	-0.4 (-1.8, 1.0)	-0.4 (-2.0, 0.6)	-1.7 (-2.6, -0.4)	0.01
BMI z-score ≤-2	55 (25.5)	19 (20.2)	23 (25.8)	13 (39.4)	0.09
**Personal medical history**					
History of TB	10 (4.5)	4 (4.2)	4 (4.3)	2 (6.1)	0.82
**Clinical and chest radiography characteristics**					
At least one clinical sign or symptom consistent with TB	175 (78.8)	63 (65.6)	88 (94.6)	24 (72.7)	<0.001
Fever	9 (4.1)	2 (2.1)	6 (6.5)	1 (3.0)	0.30
Failure to thrive	25 (20.3)	6 (10.9)	12 (25.0)	7 (35.0)	0.04
Cough	156 (70.3)	56 (58.3)	81 (87.1)	19 (57.6)	<0.001
Dyspnea	34 (15.3)	9 (9.4)	19 (20.4)	6 (18.2)	0.09
Night sweat	8 (3.6)	4 (4.2)	2 (2.2)	2 (6.1)	0.44
Chest pain	25 (11.3)	7 (7.3)	15 (16.1)	3 (9.1)	0.15
Hemoptysis	7 (3.2)	4 (4.2)	3 (3.2)	0 (0.0)	0.68
Neck pain or stiffness	14 (6.3)	8 (8.3)	5 (5.4)	1 (3.0)	0.60
Headache	1 (0.5)	1 (1.0)	0 (0.0)	0 (0.0)	1.00
TB-CXR					<0.001
No	84 (37.8)	72 (75.0)	4 (4.3)	8 (24.2)	
Yes	111 (50.0)	6 (6.3)	87 (93.5)	18 (54.5)	
Not done	27 (12.2)	18 (18.8)	2 (2.2)	7 (21.2)	
Length of stay (days), median (IQR)	9.0 (6.0, 13.0)	7.0 (4.0, 10.0)	9.0 (7.0, 13.0)	14.0 (7.0, 17.0)	<0.001
**Laboratory parameters**					
HIV result					0.01
Negative	61 (27.5)	25 (26.0)	20 (21.5)	16 (48.5)	
Positive	0 (0.0)	0 (0.0)	0 (0.0)	0 (0.0)	
Unknown/Not done	161 (72.5)	71 (74.0)	73 (78.5)	17 (51.5)	
QFT-Plus result					<0.001
Negative	176 (79.3)	87 (90.6)	74 (79.6)	15 (45.5)	
Positive	45 (20.3)	9 (9.4)	18 (19.4)	18 (54.5)	
Indeterminate	1 (0.4)	0 (0)	1 (1.0)	0 (0)	
Sputum AFB Smear					<0.001
Negative	31 (14.0)	10 (10.4)	18 (19.4)	3 (9.1)	
Positive	6 (2.7)	0 (0.0)	1 (1.1)	5 (15.2)	
Unknown/Not done	185 (83.3)	86 (89.6)	74 (79.6)	25 (75.8)	
Sputum culture					<0.001
Negative	143 (64.4)	66 (68.8)	67 (72.0)	10 (30.3)	
Positive	18 (8.1)	0 (0.0)	0 (0.0)	18 (54.5)	
Unknown/Not done	61 (27.5)	30 (31.3)	26 (28.0)	5 (15.2)	
GenXpert					<0.001
Negative	162 (73.0)	71 (74.0)	72 (77.4)	19 (57.6)	
Positive	9 (4.1)	0 (0.0)	0 (0.0)	9 (27.3)	
Unknown/Not done	51 (23.0)	25 (26.0)	21 (22.6)	5 (15.2)	
Biopsy done (both pulmonary and EPTB samples)	55 (24.8)	26 (27.1)	11 (11.8)	18 (54.5)	<0.001
Biopsy results (both pulmonary and EPTB samples)					<0.001
Negative	40 (18.0)	26 (27.1)	11 (11.8)	3 (9.1)	
Positive	15 (6.8)	0 (0.0)	0 (0.0)	15 (45.5)	
Not done	167 (75.2)	70 (72.9)	82 (88.2)	15 (45.5)	

Values are in frequency and % unless otherwise indicated; IQR, interquartile range; TB, tuberculosis; TB-CXR, chest radiograph abnormalities consistent to TB; BCG, Bacille Calmette-Guérin; BMI, body mass index; AFB, Acid-Fast Bacilli; EPTB, extra-pulmonary TB.

### Sensitivity of QFT-Plus in children with confirmed TB

Table 2 presents the patient characteristics of 33 children having confirmed TB. Compared with QFT-Plus (-) patients, patients who had a positive QFT-Plus result were likely to be older, have abnormal chest radiograph and a positive *Mtb* culture. Notably, all 9 patients who had positive Xpert results also had a positive QFT-Plus result. Stratification by specific TB site indicated that 17 out of 26 (94.4%) patients having pulmonary TB had positive QFT-Plus result while only 2 patients with extrapulmonary TB had QFT-Plus (+). The sensitivity of the QFT-Plus assay was evaluated in patients with confirmed TB (*n* = 33) and presented in Table 3. In the subgroup of patients with confirmed PTB only, the QFT-Plus assay had a sensitivity of 84.2% (CI 60.4, 96.6). Sensitivity of the QFT-Plus assay was low in the subgroups of exclusive EPTB and concomitant PTB plus EPTB with the same sensitivity of 14.3% (CI 0.36, 57.9). The overall sensitivity of the QFT-Plus assay (regardless of having PTB or EPTB) was 54.5% (CI 36.4, 71.9).

**Table 2:** Characteristics of 33 confirmed TB patients, stratified by QFT-Plus results.

Characteristics	Total	QFT-Plus (-)	QFT-Plus (+)	p-value
	(*n* = 33)	(*n* = 15)	(*n* = 18)	
Age (years), median (IQR)	9.8 (2.3, 13.4)	6.5 (2.2, 10.4)	11.5 (2.3, 13.8)	0.08
Age (years)				0.20
0–5	12 (36.4)	7 (46.7)	5 (27.8)	
6–10	6 (18.2)	4 (26.7)	2 (11.1)	
11–15	13 (39.4)	4 (26.7)	9 (50.0)	
16–17	2 (6.1)	0 (0.0)	2 (11.1)	
Male gender	17 (51.5)	8 (53.3)	9 (50.0)	0.85
BMI z-score ≤-2	13 (39.4)	6 (40.0)	7 (38.9)	0.95
History of TB	2 (6.1)	1 (6.7)	1 (5.6)	0.89
At least one clinical sign or symptom consistent with TB	24 (72.7)	9 (60.0)	15 (83.3)	0.13
Chest radiograph consistent with TB				0.01
No	8 (24.2)	5 (33.3)	3 (16.7)	
Yes	18 (54.5)	4 (26.7)	14 (77.8)	
Not done	7 (21.2)	6 (40.0)	1 (5.6)	
Sputum AFB Smear				0.09
Negative	3 (9.1)	0 (0.0)	3 (16.7)	
Positive	5 (15.2)	1 (6.7)	4 (22.2)	
Unknown/Not done	25 (75.8)	14 (93.3)	11 (61.1)	
Sputum culture				<0.001
Negative	10 (30.3)	9 (60.0)	1 (5.6)	
Positive	18 (54.5)	3 (20.0)	15 (83.3)	
Unknown/Not done	5 (15.2)	3 (20.0)	2 (11.1)	
Biopsy results (both pulmonary and EPTB samples)				<0.001
Negative	3 (9.1)	1 (6.7)	2 (11.1)	
Positive	15 (45.5)	13 (86.7)	2 (11.1)	
Unknown/Not done	15 (45.5)	1 (6.7)	14 (77.8)	
GenXpert				0.004
Negative	19 (57.6)	11 (73.3)	8 (44.4)	
Positive	9 (27.3)	0 (0.0)	9 (50.0)	
Unknown/Not done	5 (15.2)	4 (26.7)	1 (5.6)	
TB site category				<0.001
Exclusive EPTB	7 (21.2)	6 (40.0)	1 (5.6)	
Concomitant PTB and EPTB	7 (21.2)	6 (40.0)	1 (5.6)	
Exclusive PTB	19 (57.6)	3 (20.0)	16 (88.9)	
Specific TB site (patient may have multiple lesion sites)				
Pulmonary	26 (78.8)	9 (60.0)	17 (94.4)	0.02
Lymph nodes	9 (27.3)	7 (46.7)	2 (11.1)	0.02
Bone	4 (12.1)	4 (26.7)	0 (0.0)	0.02
Pleural	1 (3.0)	1 (6.7)	0 (0.0)	0.27

Values are in frequency and % unless otherwise indicated; IQR, interquartile range; TB, tuberculosis; BCG, Bacille Calmette-Guerin; BMI, body mass index; AFB, Acid- Fast Bacilli; PTB, pulmonary TB; EPTB, extra-pulmonary TB

**Table 3:** Sensitivity of QFT plus assay in children with confirmed TB (*n* = 33).

	Total number of confirmed TB with data available	Number of QFT-Plus (+)	Sensitivity, %
			(95% CI)
All confirmed TB (*n* = 33)	** **	** **	** **
Overall (regardless PTB or EPTB)	33	18	54.5 (36.4, 71.9)
Exclusive PTB	19	16	84.2 (60.4, 96.6)
Exclusive EPTB	7	1	14.3 (0.36, 57.9)
Concomitant PTB and EPTB	7	1	14.3 (0.36, 57.9)
Confirmed TB and age ≤5 years (*n* = 12)	** **	** **	** **
Overall (regardless PTB or EPTB)	12	5	41.7 (15.2, 72.3)
Exclusive PTB	5	5	100 (47.8, 100)
Exclusive EPTB	3	0	0.0 (0.0, 70.8)
Concomitant PTB and EPTB	4	0	0.0 (0.0, 60.2)
Confirmed TB and age 6–17 years (*n* = 21)	** **	** **	** **
Overall (regardless PTB or EPTB)	21	13	61.9 (38.4, 81.9)
Exclusive PTB	14	11	78.6 (49.2, 95.3)
Exclusive EPTB	4	1	25.0 (0.63, 80.6)
Concomitant PTB and EPTB	3	1	33.33 (0.84, 90.6)

AFB, Acid- Fast Bacilli; TB, tuberculosis; PTB, pulmonary TB; EPTB, extra-pulmonary TB; TB-CXR, chest radiography abnormalities consistent to TB; CI, confidence interval. Older children (6–17 years) were group together given the small size if they are stratified by 5-year age group.

In 12 confirmed TB patients who were <5 years of age, QFT-Plus detected 5/5 exclusive PTB cases (sensitivity of 100% [95% CI 47.8, 100]), while the assay had discordant results for all 3 cases of exclusive EPTB and 4 cases of concomitant PTB and EPTB. In 21 confirmed TB patients age 6–17 years, the sensitivity of QFT-Plus in the PTB, EPTB and concomitant EPTB and PTB was 78.6%, 25.0% and 33.3%, respectively (Table 3).

### Quantitative QFT-Plus results and agreement between TB1 and TB2

S1 Table presents the quantitative measurements of the QFT-Plus results. Compared with patients in the exclusive EPTB and concomitant EPTB+PTB groups, patients with exclusive PTB had higher median values of TB1-Nil (p = 0.004) and TB2-Nil (p = 0.01). Among 45 positive QFT-Plus results (Fig 1), 37 were identified by both TB1 and TB2, seven identified by TB2 only and one identified by TB1 only. The percent agreement between TB1 and TB2 was 96.4% (95% CI 93.9, 98.9) with a Cohen’s *Kappa* efficient of 0.88 (95% CI 0.80, 0.96).

### Characteristics associated with QFT-Plus results

The association of patient characteristics and QFT-Plus results were also evaluated on 221 out of 222 patients who were QFT-Plus negative (-) or positive (+). Only one patient who had an indeterminate QFT-Plus result was excluded from this specific analysis. The crude associations between QFT-Plus (+) and patient characteristics are presented in S2 Table. Compared with QFT-Plus (-) patients, patients who had a positive QFT-Plus result were significantly older (median 9.2 years; IQR 2.8, 13.4 versus 5.0 years; IQR 2.2, 10.4; p = 0.01) and had a higher proportion of TB history (11.1% versus 2.9%; p = 0.03) and TB-CXR (66.7% versus 45.5%, p = 0.01). Multiple logistic regression modeling showed that history of TB (aOR 6.30; CI 1.34, 29.75; p = 0.02), TB-CXR (aOR 2.61; CI 1.04, 6.53; p = 0.04) and confirmed TB (aOR 6.47; CI 2.48, 16.85; p<0.001) were significantly associated with having a positive QFT-Plus result with an area under the ROC curve of 0.77 (S2 Table).

## Discussion

Although IGRAs have been recommended to detect TB infection among children 2 years or older by the American Academy of Pediatrics and children 5 years or older by the American Thoracic Society, Infectious Diseases Society of America, and Centers for Disease Control and Prevention, little information is available regarding the use of QFT-Plus, the newest IGRA, in pediatric populations [24, 25]. Our study is a preliminary attempt to evaluate the performance of QFT-Plus in children from two months to 17 years of age. The overall sensitivity of QFT-Plus in our study (54.5%) is comparable with the sensitivity of QFT-Gold In-Tube (QFT-GIT), QFT-Plus’s predecessor, as reported by meta-analysis in pediatric populations [26]. QFT-Plus had a rather good sensitivity in pediatric patients with exclusive PTB. Although all the children <5 years of age who had confirmed PTB were detected by QFT-Plus (i.e. sensitivity of 100%), this result should be interpreted cautiously given the small number of children in this group (*n* = 5). The sensitivity of 84.2% found in our study for patients with exclusive PTB is comparable with the sensitivity of 87.9% (95 out of 116) described by Barcellini et al. in a multi-center study in Europe for adults with bacteriologically confirmed TB [27]. Kosloff et al. also suggested a similar sensitivity of 83% (CI 75, 90) in Zambian adults with active tuberculosis [16]. Meanwhile, Takasaki et al. reported a much higher sensitivity of 98.9% (CI 93.4, 99.8) in Japanese adults with laboratory-confirmed active TB [15].

The QFT-Plus sensitivity was poor in pediatric patients with exclusive EPTB and concomitant PTB and EPTB. Although data on the diagnostic performance of QFT-Plus on EPTB are still limited, there are inconsistent sensitivities of QFT-GIT reported by different studies in adults, which range from 38.5% to 81.5% depending on the extra-pulmonary TB site [28, 29]. In a recent study on the use of the QFT-GIT in adults, Kim et al. also confirmed the variation of false-negative QFT-GIT depending on the site of EPTB with nearly 30% of all EPTB cases having a false-negative QFT-GIT result [30].

In our study, the positivity of QFT-Plus appeared to increase with older age with significant difference in univariate analysis and a trend toward significance in multiple logistic regression analysis. This finding is consistent with an observation reported by other authors that a higher rate of false-negative IGRA results in young children [31–33]. A longer time of exposure or repeated exposure to *Mtb* may play a role in increasing the odds of having a positive QFT-Plus result in patients with a history of TB compared to patients who had the first episode of TB disease. In a study on adults with TB contacts by Barcellini et al., the increased TB contact time was found significantly associated with higher odds of having a positive QFT-Plus [27].

Although most of the QFT-Plus positive results were identified by both TB1 and TB2 responses, a higher positivity proportion was found with the TB2 antigenic response. Studies in adults reported similar findings [34, 35]. Petruccioli et al. also observed an association of the TB2 response with active TB and severe TB disease which indicates that TB2 stimulation induces a CD8 T-cell response in absence of a CD4-response [34].

Our study has several limitations. First, only 30.1% (222/738) of all pediatric patients seen by physicians at NLH from April through December 2017 had the QFT-Plus assay done. As the QFT-Plus was not part of the National TB Program’s diagnostic arsenal, some parents may not have wanted their child to be phlebotomized or tested, especially when the parent had to cover the cost of the QFT-Plus assay themselves. Given that the data for patients who did not have a QFT-Plus result were unavailable, our study cannot rule out completely classification bias. Additionally, this is a single-center retrospective study with little information on the referral pattern. Although our findings may not be generalizable to other populations, the study results can be used in designing larger studies, especially multi-center prospective studies which would include patients with different socioeconomic backgrounds. As the tuberculin skin test (TST) is no longer used by the National TB Program in Vietnam at the time of the study, we could not compare the sensitivity of QFT-Plus with that of the TST, a commonly-used surrogate for LTBI. It was not possible to differentiate between *Mtb* and NTM in patients diagnosed by biopsy alone. However, NTM diagnosis is rare in Vietnam. Although NTM data is not available in HIV-negative TB patients, especially in children, the NTM prevalence in TB/HIV co-infected patients has been reported was in the range of 0.25% to 7% based on previous studies [36, 37]. Therefore, although we cannot rule out completely the possibility of misdiagnosis of NTM in TB patients confirmed by the biopsy alone, the effect of this possible misdiagnosis on the calculation of the QFT-Plus sensitivity, if any, would be minimal. The reason why QFT-Plus had the same sensitivity in patients with PTB + EPTB as in patients with EPTB alone cannot be completely explained by our current data and will need to be verified in future studies with larger sample sizes and more detailed information regarding the clinical and immunologic extent of PTB and EPTB as well as time from diagnosis and treatment initiation to blood sample collection for QFT-Plus. Lastly, with the lack of healthy controls, we could not completely evaluate the diagnostic performance of QFT-Plus in children such as specificity, positive and negative predictive values.

## Conclusion

Our study reports initial information on the performance of QFT-Plus in children with confirmed TB in a high TB prevalence setting. Although the QFT-Plus assay had rather good sensitivity in the subgroup of patients with exclusive PTB, the assay had poor sensitivity in EPTB patients. Our findings also indicated that the patient’s age, history of previous TB disease, and TB-related abnormalities on chest radiography may be associated with a positive QFT-Plus result.

## Supporting information

S1 Table Quantitative results of QFT-Plus assay. Older children (6–17 years) were group together given the small size if they are stratified by 5-year age group.
(DOCX)

S2 Table Characteristics associated with a QFT-Plus (+) in patients having either QFT-Plus (-) or QFT-Plus (+) results (N = 221). Values are in frequency and % unless otherwise indicated; IQR, interquartile range; TB, tuberculosis; BCG, Bacille Calmette-Guérin; BMI, body mass index; AFB, Acid- Fast Bacilli; PTB, pulmonary TB; EPTB, extra-pulmonary TB. One patient having indeterminate QFT-Plus were excluded from this analysis. Area under ROC curve: 0.77; TB, tuberculosis; BMI, body mass index. Model was run on N = 214 patients having complete data for all variables used in the model.
(DOCX)

S1 Dataset (XLSX)
