# Supplementary material for: Sensitivity and characteristics associated with positive QuantiFERON-TB Gold-Plus assay in children with confirmed tuberculosis
Source: PLoS One. 2019 Mar 4;14(3):e0213304. doi: 10.1371/journal.pone.0213304 (PMC6398855; doi:10.1371/journal.pone.0213304)
Supplement: S1 Table — Older children (6–17 years) were group together given the small size if they are stratified by 5-year age group. (DOCX) [file pone.0213304.s001.docx]

**S1 Table. Quantitative results of QFT-Plus assay**

| **All patients (N=222)** | **All patients** | **Unlikely TB** | **Unconfirmed TB** | **Confirmed TB** | **Overall** |
| --- | --- | --- | --- | --- | --- |
|  | **(N=222)** | **(*n*=96)** | **(*n*=93)** | **(*n*=33)** | **p-value** |
| Nil, median (IQR) | 0.1 (0.1, 0.3) | 0.1 (0.1, 0.2) | 0.1 (0.1, 0.2) | 0.2 (0.1, 0.9) | 0.11 |
| TB1, median (IQR) | 0.1 (0.1, 0.5) | 0.1 (0.1, 0.2) | 0.1 (0.1, 0.6) | 0.7 (0.1, 4.1) | <0.001 |
| TB1-Nil, median (IQR) | 0.0 (-0.0, 0.1) | 0.0 (-0.0, 0.0) | 0.0 (-0.0, 0.1) | 0.1 (0.0, 3.5) | 0.002 |
| TB2, median (IQR) | 0.1 (0.1, 0.7) | 0.1 (0.1, 0.3) | 0.1 (0.1, 0.6) | 1.3 (0.1, 4.5) | <0.001 |
| TB2-Nil, median (IQR) | 0.0 (-0.0, 0.2) | 0.0 (-0.0, 0.0) | 0.0 (-0.0, 0.1) | 0.2 (0.0, 3.8) | 0.001 |
| **All confirmed TB (n=33)** | **Total** | **Exclusive EPTB** | **Concomitant PTB and EPTB** | **Exclusive PTB** | **Overall** |
|  | **(*n*=33)** | **(*n*=7)** | **(*n*=7)** | **(*n*=19)** | **p-value** |
| Nil, median (IQR) | 0.2 (0.1, 0.9) | 0.1 (0.1, 0.2) | 0.2 (0.1, 1.2) | 0.3 (0.1, 0.9) | 0.50 |
| TB1, median (IQR) | 0.7 (0.1, 4.1) | 0.1 (0.1, 0.2) | 0.2 (0.1, 4.8) | 1.8 (0.3, 6.8) | 0.08 |
| TB1-Nil, median (IQR) | 0.1 (0.0, 3.5) | 0.0 (-0.1, 0.0) | 0.0 (-0.0, 0.1) | 0.9 (0.0, 4.8) | 0.004 |
| TB2, median (IQR) | 1.3 (0.1, 4.5) | 0.1 (0.1, 0.2) | 0.2 (0.1, 6.2) | 2.2 (0.5, 9.4) | 0.09 |
| TB2-Nil, median (IQR) | 0.2 (0.0, 3.8) | 0.0 (-0.1, 0.1) | 0.0 (-0.0, 0.1) | 2.0 (0.2, 7.7) | 0.01 |
| **Confirmed TB and age ≤5 years (n=12)** | **Total** | **Exclusive EPTB** | **Concomitant PTB and EPTB** | **Exclusive PTB** | **Overall** |
|  | **(*n*=12)** | **(*n*=3)** | **(*n*=4)** | **(*n*=5)** | **p-value** |
| Nil, median (IQR) | 0.1 (0.1, 0.6) | 0.1 (0.1, 0.2) | 0.1 (0.1, 0.7) | 0.2 (0.1, 0.9) | 0.91 |
| TB1, median (IQR) | 0.2 (0.1, 1.2) | 0.1 (0.1, 0.1) | 0.1 (0.1, 0.7) | 1.1 (0.4, 2.2) | 0.04 |
| TB1-Nil, median (IQR) | 0.0 (0.0, 0.3) | 0.0 (-0.1, 0.0) | 0.0 (-0.0, 0.0) | 0.4 (0.3, 2.0) | 0.03 |
| TB2, median (IQR) | 0.3 (0.1, 1.7) | 0.1 (0.1, 0.1) | 0.1 (0.1, 0.7) | 2.1 (1.3, 2.2) | 0.03 |
| TB2-Nil, median (IQR) | 0.1 (0.0, 1.2) | 0.0 (-0.1, 0.0) | 0.0 (-0.0, 0.0) | 2.0 (0.4, 2.0) | 0.02 |
| **Confirmed TB and age 6-17 years (n=21)** | **Total** | **Exclusive EPTB** | **Concomitant PTB and EPTB** | **Exclusive PTB** | **Overall** |
|  | **(*n*=21)** | **(*n*=4)** | **(*n*=3)** | **(*n*=14)** | **p-value** |
| Nil, median (IQR) | 0.2 (0.1, 0.9) | 0.1 (0.1, 0.2) | 0.2 (0.0, 7.3) | 0.4 (0.1, 0.9) | 0.55 |
| TB1, median (IQR) | 1.8 (0.1, 5.5) | 0.2 (0.1, 1.5) | 4.8 (0.1, 7.2) | 2.8 (0.1, 6.8) | 0.43 |
| TB1-Nil, median (IQR) | 0.6 (0.0, 4.5) | -0.0 (-0.1, 1.4) | 0.0 (-0.1, 4.5) | 2.2 (0.0, 4.8) | 0.08 |
| TB2, median (IQR) | 2.6 (0.1, 6.7) | 0.2 (0.2, 1.4) | 6.2 (0.1, 6.7) | 4.2 (0.1, 9.4) | 0.45 |
| TB2-Nil, median (IQR) | 1.2 (0.0, 5.9) | 0.1 (-0.1, 1.3) | 0.0 (-1.0, 6.4) | 3.7 (0.0, 7.7) | 0.18 |

Older children (6-17 years) were group together given the small size if they are stratified by 5-year age group.
