# Supplementary material for: Sensitivity and characteristics associated with positive QuantiFERON-TB Gold-Plus assay in children with confirmed tuberculosis
Source: PLoS One. 2019 Mar 4;14(3):e0213304. doi: 10.1371/journal.pone.0213304 (PMC6398855; doi:10.1371/journal.pone.0213304)
Supplement: S2 Table — Values are in frequency and % unless otherwise indicated; IQR, interquartile range; TB, tuberculosis; BCG, Bacille Calmette-Guérin; BMI, body mass index; AFB, Acid- Fast Bacilli; PTB, pulmonary TB; EPTB, extra-pulmonary TB. One patient having indeterminate QFT-Plus were excluded from this analysis. Area under ROC curve: 0.77; TB, tuberculosis; BMI, body mass index. Model was run on N = 214 patients having complete data for all variables used in the model. (DOCX) [file pone.0213304.s002.docx]

**S2 Table. Characteristics associated with a QFT-Plus (+) in patients having either QFT-Plus (-) or QFT-Plus (+) results (N=221)**

| **Characteristics** | **QFT-Plus (-)** | **QFT-Plus (+)** | **Unadjusted OR** | **Adjusted OR**  **(95% CI)** | **p-value** |
| --- | --- | --- | --- | --- | --- |
|  | **(*n*=176)** | **(*n*=45)** | **(95% CI)** |  |  |
| Age (years), median (IQR) | 5.0 (2.2, 10.4) | 9.2 (2.8, 13.4) | 1.10 (1.02, 1.17) | 1.08 (0.99, 1.17) | 0.07 |
| Age (years) |  |  |  |  |  |
| 0-5 | 88 (50.0) | 17 (37.8) | (reference) |  |  |
| 6-10 | 40 (22.7) | 8 (17.8) | 1.04 (0.41, 2.60) |  |  |
| 11-15 | 46 (26.1) | 15 (33.3) | 1.69 (0.77, 3.68) |  |  |
| 16-17 | 2 (1.1) | 5 (11.1) | 12.94 (2.32, 72.28) |  |  |
| Male gender | 107 (60.8) | 26 (57.8) | 0.88 (0.45, 1.71) |  |  |
| Height (cm) | 110.0 (82.0, 137.0) | 123.0 (95.5, 147.5) | 1.01 (1.00, 1.02) |  |  |
| Weight (kg) | 17.0 (11.0, 29.0) | 24.0 (13.0, 36.0) | 1.03 (1.00, 1.05) |  |  |
| BMI (raw) | 15.9 (13.6, 18.1) | 15.5 (14.2, 18.7) | 0.98 (0.90, 1.06) |  |  |
| BMI z-score for sex and age, median (IQR) | -0.4 (-2.0, 0.9) | -1.1 (-2.1, 0.0) | 0.91 (0.79, 1.03) | 0.95 (0.81, 1.11) | 0.49 |
| BMI z-score ≤-2 | 42 (24.6) | 13 (29.5) | 1.29 (0.62, 2.69) |  |  |
| History of TB | 5 (2.9) | 5 (11.1) | 4.25 (1.17, 15.39) | 6.30 (1.34, 29.75) | 0.02 |
| At least one clinical sign or symptom consistent with TB | 136 (77.3) | 38 (84.4) | 1.60 (0.66, 3.85) | 1.67 (0.56, 5.01) | 0.36 |
| Fever | 8 (4.5) | 1 (2.2) | 0.48 (0.06, 3.92) |  |  |
| Failure to thrive | 20 (20.4) | 5 (20.8) | 1.03 (0.34, 3.09) |  |  |
| Cough | 120 (68.2) | 35 (77.8) | 1.63 (0.76, 3.53) |  |  |
| Dyspnea | 25 (14.2) | 8 (17.8) | 1.31 (0.55, 3.13) |  |  |
| Night sweat | 8 (4.5) | 0 (0.0) | -- |  |  |
| Chest pain | 18 (10.2) | 7 (15.6) | 1.62 (0.63, 4.15) |  |  |
| Hemoptysis | 6 (3.4) | 1 (2.2) | 0.64 (0.08, 5.49) |  |  |
| Neck pain or stiffness | 12 (6.8) | 2 (4.4) | 0.64 (0.14, 2.95) |  |  |
| Chest radiograph consistent with TB |  |  |  |  |  |
| No | 75 (42.6) | 9 (20.0) | (reference) | (reference) |  |
| Yes | 80 (45.5) | 30 (66.7) | 3.13 (1.39, 7.02) | 2.61 (1.04, 6.53) | 0.04 |
| Not done | 21 (11.9) | 6 (13.3) | 2.38 (0.76, 7.45) | 0.95 (0.24, 3.75) | 0.94 |
| Length of stay (days), median (IQR) | 8.5 (5.5, 12.5) | 9.0 (7.0, 13.0) | 1.01 (0.98, 1.04) |  |  |
| Sputum AFB Smear |  |  |  |  |  |
| Negative | 25 (14.2) | 6 (13.3) | (reference) | (reference) |  |
| Positive | 2 (1.1) | 4 (8.9) | 8.33 (1.23, 56.67) | 2.29 (0.26, 20.14) | 0.46 |
| Unknown/Not done | 149 (84.7) | 35 (77.8) | 0.98 (0.37, 2.57) | 1.76 (0.52, 6.03) | 0.37 |
| Confirmed TB | 15 (8.5) | 18 (40.0) | 7.16 (3.22, 15.88) | 6.47 (2.48, 16.85) | <0.001 |

Values are in frequency and % unless otherwise indicated; IQR, interquartile range; TB, tuberculosis; BCG, Bacille Calmette-Guérin; BMI, body mass index; AFB, Acid- Fast Bacilli; PTB, pulmonary TB; EPTB, extra-pulmonary TB. One patient having indeterminate QFT-Plus were excluded from this analysis. Area under ROC curve: 0.77; TB, tuberculosis; BMI, body mass index. Model was run on N=214 patients having complete data for all variables used in the model.
